# Parental employment status and preschoolers' emotional and behavioral problems in Western China: a cross-sectional study

**DOI:** 10.3389/fped.2026.1897604

**Published:** 2026-08-19

**Authors:** Guiting Ren, Hongli Sun, Jingyu Bu

**Affiliations:** 1 School of Humanities and Social Sciences, Xi’an Jiaotong University, Xi’an, Shaanxi, China; 2 Faculty of Humanities, Shangluo University, Shangluo, Shaanxi, China; 3 Xi’an Key Laboratory of Children’s Health and Diseases, Shaanxi Institute for Pediatric Diseases, Xi’an Children’s Hospital, Affiliated Children’s Hospital of Xi’an Jiaotong University, Xi’an, Shaanxi, China; 4 Department of Pediatrics, Tangdu Hospital, Fourth Military Medical University, Xi’an, Shaanxi, China

**Keywords:** a cross-sectional study, China, emotional and behavioral problems, parental employment status, preschoolers

## Abstract

**Objectives:**

Guided by the Family Economic Stress Model, this study investigated the associations between parental employment status and emotional and behavioral problems among preschoolers in Western China.

**Methods:**

This cross-sectional study included 21,968 children aged 3–6 years recruited from 189 kindergartens. Parental employment status was categorized into four family-level patterns and further analyzed separately for paternal and maternal employment. Children's outcomes were assessed using the Strengths and Difficulties Questionnaire.

**Results:**

Father-only employment was associated with higher internalizing problems (aOR = 1.11) but lower externalizing problems (aOR = 0.90); dual non-employment was associated with increased internalizing problems (aOR = 1.31). Paternal unemployment was linked to externalizing problems (aOR = 1.25), while maternal non-employment was associated with internalizing problems (aOR = 1.11–1.27). No significant effect modification was observed after FDR correction.

**Conclusions:**

Parental employment status is associated with preschoolers' emotional and behavioral problems in a gender-differentiated manner. Paternal unemployment is primarily linked to externalizing problems, whereas maternal non-employment is more strongly associated with internalizing problems.

## 1 Introduction

Emotional and behavioral problems in preschoolers represent a major public health concern worldwide, with an estimated 10%–20% of children experiencing clinically significant difficulties that can persist into later life (1, 2). The preschool period (3–6 years) is a critical window for neurodevelopment and socialization, during which family environmental factors profoundly shape long-term psychosocial outcomes (3, 4). In China, rapid socioeconomic transformation over the past four decades has dramatically reshaped family structures and parental employment patterns (5, 6). Maternal labor force participation remains high, while paternal roles have increasingly shifted toward shared caregiving responsibilities (7, 8). These changes, coupled with the recent relaxation of fertility policies, have created complex family dynamics that may influence children's emotional and behavioral development (9). Western China presents a particularly compelling context for examining these dynamics. As a region that has historically experienced slower economic development compared to the eastern coastal areas, Western China has undergone rapid yet uneven socioeconomic transitions under the national Western Development Strategy. These regional disparities have produced distinctive family employment patterns, including higher rates of informal labor market participation, greater exposure to economic volatility, and more pronounced urban–rural socioeconomic gradients. Furthermore, preschool mental health services and family support infrastructure in Western China remain less developed than in eastern regions, making early identification of risk factors especially critical. Despite these unique contextual features, large-scale epidemiological evidence on preschoolers' emotional and behavioral problems in Western China remains scarce, with existing studies predominantly concentrated in affluent eastern cities (10). Conducting this research in Western China therefore fills a critical geographic gap and provides essential evidence for tailoring family support policies to the specific needs of less-developed regions.

Guided by the Family Economic Stress Model, which posits that financial hardship-induced parental stress impairs child adjustment through disrupted family processes (11, 12), extensive research has demonstrated associations between parental unemployment and adverse child mental health outcomes (13–16). However, the literature has three critical gaps. First, existing evidence predominantly comes from Western contexts; large-scale studies in non-Western settings such as China remain limited. Second, most research has focused on maternal employment or aggregated parental employment, without disentangling the independent and combined effects of paternal and maternal employment status on child outcomes (17)—a distinction particularly relevant given evolving gender roles in contemporary China. Third, studies have typically used global measures of child behavior, failing to distinguish between internalizing problems, externalizing problems, and prosocial behavior, which may have distinct etiological pathways (18, 19).

To address these gaps, this study leveraged a large, representative sample of 21,968 preschoolers from Western China to investigate the associations between parental employment status and children's emotional and behavioral problems, guided by the Family Economic Stress Model. Specifically, we examined: (1) the associations between family employment patterns and child psychosocial outcomes; (2) the independent effects of paternal and maternal employment status; and (3) the moderating role of sociodemographic factors. We hypothesized that parental non-employment would be associated with increased risks of child difficulties, with distinct patterns for internalizing vs. externalizing problems. These findings aim to provide evidence-based insights for family support policies and interventions in the context of China's evolving socioeconomic landscape.

## 2 Methods

### 2.1 Participants

This cross-sectional study was conducted in collaboration with the local educational authorities in Yan'an, a prefecture-level city in northern Shaanxi Province, Western China. Yan'an was selected for several methodological and contextual considerations. First, as a renowned revolutionary base area and a representative city of the Loess Plateau region, Yan'an exemplifies the socioeconomic profile of medium-sized cities in Western China that have experienced rapid yet uneven development following national poverty alleviation and Western Development strategies. Its economy, historically reliant on agriculture and energy resources, is undergoing structural transformation, producing distinctive patterns of parental employment that include substantial informal labor participation and exposure to economic volatility. Second, with a permanent resident population of approximately 2.2 million and a relatively lower urbanization rate compared to major provincial capitals in Western China, Yan'an captures the demographic heterogeneity—encompassing both urban districts and vast rural–urban fringe areas—characteristic of less-developed western regions. Third, despite its historical significance, large-scale epidemiological research on preschool mental health remains virtually absent in Yan'an, making it an ideal setting to fill the geographic evidence gap for non-core cities in Western China. Finally, the established collaborative relationships with local educational authorities and the standardized kindergarten system in Yan'an facilitated the implementation of large-scale, community-based data collection. Between February 28 and March 5, 2025, a stratified cluster sampling design was employed to recruit a representative sample of children aged 3–6 years from 189 kindergartens in Yan'an. All children and their parents from the selected kindergartens were invited to participate, and one parent per child completed a self-administered paper-based questionnaire. A total of 25,017 valid questionnaires were returned and included in the initial screening.

After data cleaning, 2,408 respondents who were not the biological parents of the target child were excluded. Among the remaining 22,609 parent respondents, we further excluded 641 cases with implausible sex concordance between spouses (i.e., both partners reported as male or both as female) to ensure accurate identification of maternal and paternal characteristics, yielding a final analytic sample of 21,968 parent–child dyads.

Missing data on child age, paternal age, and maternal age were minimal, with proportions of 0.08%, 3.05%, and 3.85%, respectively—all well below the conventional threshold of 5%. Rather than excluding these cases listwise, which would have introduced selection bias and reduced statistical power, we retained all participants in the primary analyses and addressed missingness using multiple imputation by chained equations (MICE) with five imputations under the missing-at-random assumption (Supplementary Table 3). This approach allowed us to preserve the full analytic sample while appropriately accounting for the uncertainty inherent in imputing missing values. Sensitivity analyses comparing the results from the imputed and complete-case datasets yielded consistent findings, confirming the robustness of our conclusions.

### 2.2 Variables

#### 2.2.1 Parental employment status

Parental employment status was assessed based on the respondent's occupation at the time of the survey. For the primary analysis, respondents were categorized into four mutually exclusive groups according to the combined employment status of both parents: (1) dual-earner households, in which both parents were employed; (2) father-only employed households, in which the father was employed while the mother was not in the labor force or unemployed; (3) mother-only employed households, in which the mother was employed while the father was not in the labor force or unemployed; and (4) dual non-employment households, in which neither parent was employed. For the secondary analyses examining the independent effects of paternal and maternal employment, each parent's employment status was classified into three categories: employed, not in the labor force (including homemakers and retirees), and unemployed.

#### 2.2.2 Preschoolers' emotional and behavioral problems

To assess children's emotional and behavioral problems, this study used the official simplified Chinese version of the Strengths and Difficulties Questionnaire (SDQ)—a relatively concise tool developed by R. Goodman in 1997 to screen for psychosocial issues in children aged 4–17 (20). When applied to multidimensional behavioral assessment for younger age groups, the validity and reliability of the total difficulty score in the parent-reported SDQ have been verified to be adequate; specifically, the Cronbach's alpha coefficients for all SDQ dimensions ranged from 0.620 to 0.874. Additionally, this tool is well-suited for both screening practices and scientific research (21, 22).

The SDQ comprises 25 items, divided into 5 subscales: emotional symptoms (e.g., frequently feeling unhappy or downhearted), conduct problems (e.g., getting into fights with other children), hyperactivity/inattention (e.g., constantly fidgeting or squirming), peer relationship problems (e.g., preferring to play alone), and prosocial behavior (e.g., being considerate of others' feelings). Parents or guardians rated each item on a 0–1–2 scale, which correspond to “not true,” “moderately true,” and “definitely true,” respectively.

Risk thresholds were defined as follows: internalizing problems were classified as at-risk if emotional symptom scores exceeded 3; externalizing problems were marked as at-risk if conduct problem scores over 2; total difficulty scores above 14 were considered at-risk; and prosocial behavior scores below 6 were regarded as abnormal. Notably, the total difficulty score was computed without including the prosocial behavior subscale. In line with Goodman's classification criteria (23), scores for each subscale (ranging from 0 to 10) and total difficulty scores (ranging from 0 to 40) were categorized into either normal or at-risk groups.

#### 2.2.3 Covariates

Covariates included in the analyses were: child age (continuous, years), child gender (boys vs. girls), marital status (married/living with partner vs. others), annual family income (≤30, >30 to 100, >100 thousand RMB), CES-D scores (continuous), parental age (continuous, years), paternal and maternal educational attainment (junior high school or below, high school diploma/junior college, undergraduate degree or above), paternal and maternal employment status (employed, not in the labor force, unemployed), and paternal and maternal smoking status and alcohol consumption (yes vs. no). In the regression models, child age, parental age, and CES-D scores were treated as continuous variables to retain maximum information and statistical power. For stratified analyses, child age was categorized as <4, 4–5, and ≥5 years; paternal age as <30, 30–44, and ≥45 years; maternal age as <30, 30–44, and ≥45 years; and CES-D scores were dichotomized as <16 (normal) vs. ≥16 (at risk of depression).

### 2.3 Statistical analysis

All statistical analyses were performed using R software (version 4.5.1; R Foundation for Statistical Computing, Vienna, Austria). Baseline characteristics of the study population were summarized according to family employment status. Continuous variables were presented as means with standard deviations (SD), and categorical variables as frequencies with percentages. Group differences were compared using the Kruskal–Wallis rank-sum test for continuous variables and the chi-square test or Fisher's exact test for categorical variables, as appropriate.

Logistic regression models were fitted to examine the associations between employment status and child psychosocial outcomes. Three sets of analyses were conducted: (1) family employment status (four categories: dual-earner, father-only employed, mother-only employed, and dual non-employment); (2) paternal employment status (employed, not in labor force, unemployed), adjusted for maternal employment status; and (3) maternal employment status (employed, not in labor force, unemployed), adjusted for paternal employment status. For each exposure, two sequential models were constructed: Model 1 was unadjusted, and Model 2 was adjusted for all covariates, including child age and gender, marital status, annual family income, CES-D scores, parental age, and paternal and maternal educational attainment, smoking status, and alcohol consumption. Results were reported as odds ratios (ORs) with 95% confidence intervals (CIs).

To explore potential effect modification, stratified analyses were performed across subgroups defined by child age, child gender, marital status, annual family income, depression risk, paternal age, paternal education, paternal smoking, paternal alcohol consumption, maternal age, maternal education, maternal smoking, and maternal alcohol consumption. Within each stratum, logistic regression models were fitted with adjustment for all other stratification variables except the one being stratified. Interaction terms were formally tested by including cross-product terms in the regression models. To control for multiple testing in subgroup analyses, the Benjamini–Hochberg false discovery rate (FDR) method was applied.

Multicollinearity among covariates in the fully adjusted models was assessed using variance inflation factors (VIFs), with VIF values below 5 considered indicative of no significant multicollinearity. Given the low proportions of missing data in age variables (child age: 0.08%; paternal age: 3.05%; maternal age: 3.85%), multiple imputation by chained equations (MICE) with five imputations was performed under the missing-at-random assumption to address missingness while preserving the full analytic sample. Logistic regression models were fitted to each imputed dataset, and results were pooled using Rubin's rules. Sensitivity analyses comparing the imputed results with complete-case analyses were conducted to confirm the robustness of the findings. A two-sided *P*-value <0.05 was considered statistically significant.

## 3 Results

### 3.1 Characteristics of included participants

A total of 21,968 participants were included in the final analysis. The study population consisted of 14,835 (67.5%) dual-earner households, 5,994 (27.3%) father-only employed households, 203 (0.9%) mother-only employed households, and 936 (4.3%) dual non-employment households.

Significant differences were observed across the four groups in child age, parental age, CES-D scores, marital status, family income, parental education, parental smoking and maternal alcohol consumption, and all SDQ outcomes (all *P* < 0.05). No significant differences were found in child gender (*P* = 0.746) or paternal alcohol consumption (*P* = 0.992). Children from dual non-employment households consistently showed the highest prevalence of psychosocial difficulties, while those from dual-earner households had the lowest rates across all SDQ subscales. More details are presented in Table 1.

**Table 1:** Demographic and clinical characteristics of the study population by family employment status.

Variables	Dual-earner households	Father-only employment	Mother-only employment	Dual non-employment	*P*-value
	Mean + SD/*N* (%)	Mean + SD/*N* (%)	Mean + SD/*N* (%)	Mean + SD/*N* (%)	
*N*	14,835 (67.5)	5,994 (27.3)	203 (0.9)	936 (4.3)	
Child age, years	4.80 (0.88)	4.82 (0.89)	4.92 (0.85)	4.97 (0.88)	<0.001
Child gender					
Boys	7,606 (51.3)	3,094 (51.6)	111 (54.7)	487 (52.0)	0.746
Girls	7,229 (48.7)	2,900 (48.4)	92 (45.3)	449 (48.0)	
Paternal age, years	35.92 (4.51)	35.82 (4.82)	36.61 (5.61)	37.65 (5.55)	<0.001
Maternal age, years	34.54 (4.28)	33.89 (4.69)	34.96 (5.04)	35.56 (5.58)	<0.001
CES-D scores	10.70 (6.37)	12.06 (6.80)	14.36 (7.76)	14.42 (7.92)	<0.001
Marital status					
Married/living with partner	14,439 (97.3)	5,849 (97.6)	179 (88.2)	872 (93.2)	<0.001
Others	396 (2.7)	145 (2.4)	24 (11.8)	64 (6.8)	
Annual family income, in thousands, RMB					
<30	5,013 (33.8)	3,098 (51.7)	136 (67.0)	741 (79.2)	<0.001
≥30 to 100	6,171 (41.6)	2,373 (39.6)	58 (28.6)	175 (18.7)	
≥100	3,651 (24.6)	523 (8.7)	9 (4.4)	20 (2.1)	
Paternal education level					
≤Junior high school	2,947 (19.9)	3,122 (52.1)	102 (50.2)	699 (74.7)	<0.001
High school diploma and junior college	7,300 (49.2)	2,450 (40.9)	90 (44.3)	217 (23.2)	
≥Undergraduate degree	4,588 (30.9)	422 (7.0)	11 (5.4)	20 (2.1)	
Maternal education level					
≤Junior high school	2,502 (16.9)	3,082 (51.4)	61 (30.0)	659 (70.4)	<0.001
High school diploma and junior college	6,517 (43.9)	2,611 (43.6)	98 (48.3)	253 (27.0)	
≥Undergraduate degree	5,816 (39.2)	301 (5.0)	44 (21.7)	24 (2.6)	
Paternal smoking status					
No	7,570 (58.7)	3,012 (56.5)	98 (58.0)	495 (60.2)	0.027
Yes	5,329 (41.3)	2,322 (43.5)	71 (42.0)	327 (39.8)	
Maternal smoking status					
No	12,871 (88.1)	5,375 (92.1)	169 (86.7)	818 (90.7)	<0.001
Yes	1,745 (11.9)	459 (7.9)	26 (13.3)	84 (9.3)	
Paternal alcohol intake status					
No	9,040 (68.8)	3,831 (69.0)	127 (68.3)	595 (68.9)	0.992
Yes	4,102 (31.2)	1,722 (31.0)	59 (31.7)	269 (31.1)	
Maternal alcohol intake status					
No	13,027 (90.1)	5,376 (92.9)	173 (89.6)	835 (92.4)	<0.001
Yes	1,424 (9.9)	410 (7.1)	20 (10.4)	69 (7.6)	
Total difficulties					
No	12,302 (82.9)	4,681 (78.1)	144 (70.9)	673 (71.9)	<0.001
Yes	2,533 (17.1)	1,313 (21.9)	59 (29.1)	263 (28.1)	
Internalizing problems					
No	12,017 (81.0)	4,522 (75.4)	150 (73.9)	635 (67.8)	<0.001
Yes	2,818 (19.0)	1,472 (24.6)	53 (26.1)	301 (32.2)	
Externalizing problems					
No	11,265 (75.9)	4,550 (75.9)	136 (67.0)	656 (70.1)	<0.001
Yes	3,570 (24.1)	1,444 (24.1)	67 (33.0)	280 (29.9)	
Prosocial behavior					
No	7,223 (48.7)	3,068 (51.2)	107 (52.7)	503 (53.7)	<0.001
Yes	7,612 (51.3)	2,926 (48.8)	96 (47.3)	433 (46.3)	

Continuous variables presented as Mean ± SD; Categorical variables presented as *N* (%). SD, standard deviation; *N*, number of participants.

### 3.2 Associations between parental employment status and preschoolers' emotional and behavioral problems

Table 2 presents the associations between family employment status and preschoolers' emotional and behavioral problems. In the unadjusted models, compared with dual-earner households, father-only employment, mother-only employment, and dual non-employment were all significantly associated with higher odds of total difficulties and internalizing problems (all *P* < 0.05). For externalizing problems, mother-only employment (OR = 1.55, 95% CI: 1.15–2.08, *P* = 0.003) and dual non-employment (OR = 1.35, 95% CI: 1.16–1.56, *P* < 0.001) were associated with significantly higher odds, whereas father-only employment showed no significant association (OR = 1.00, 95% CI: 0.93–1.07, *P* = 0.968). For prosocial behavior, father-only employment (OR = 0.90, 95% CI: 0.85–0.96, *P* < 0.001) and dual non-employment (OR = 0.82, 95% CI: 0.72–0.93, *P* = 0.003) were associated with lower odds of abnormality, while mother-only employment was not significantly associated (OR = 0.85, 95% CI: 0.64–1.12, *P* = 0.256). After adjusting for all covariates, most of these associations were substantially attenuated. Father-only employment remained significantly associated with increased odds of internalizing problems (adjusted OR = 1.11, 95% CI: 1.02–1.21, *P* = 0.017) and decreased odds of externalizing problems (adjusted OR = 0.90, 95% CI: 0.83–0.98, *P* = 0.013). Dual non-employment was associated with higher odds of internalizing problems (adjusted OR = 1.31, 95% CI: 1.10–1.55, *P* = 0.002). No significant associations were observed for mother-only employment across any outcome in the fully adjusted models.

**Table 2:** Association between parental employment status and preschoolers' emotional and behavioral problems.

Variables	Non-adjusted model	Adjusted model
	OR (95% CI) *P* value	OR (95% CI) *P* value
Total difficulties		
Dual-earner households	1.0	1.0
Father-only employment	1.36 (1.26–1.47) 0.0000	1.02 (0.93–1.12) 0.6545
Mother-only employment	1.99 (1.46–2.69) 0.0000	1.21 (0.85–1.7) 0.2752
Dual non-employment	1.9 (1.63–2.2) 0.0000	1.08 (0.9–1.29) 0.4032
Internalizing problems		
Dual-earner households	1.0	1.0
Father-only employment	1.39 (1.29–1.49) 0.0000	1.11 (1.02–1.21) 0.0166
Mother-only employment	1.51 (1.09–2.05) 0.0110	0.97 (0.68–1.37) 0.8758
Dual non-employment	2.02 (1.75–2.33) 0.0000	1.31 (1.1–1.55) 0.0018
Externalizing problems		
Dual-earner households	1.0	1.0
Father-only employment	1 (0.93–1.07) 0.9682	0.9 (0.83–0.98) 0.0126
Mother-only employment	1.55 (1.15–2.08) 0.0034	1.2 (0.87–1.65) 0.2551
Dual non-employment	1.35 (1.16–1.56) 0.0001	1.05 (0.89–1.24) 0.5383
Prosocial behavior		
Dual-earner households	1.0	1.0
Father-only employment	0.9 (0.85–0.96) 0.0001	1.01 (0.94–1.09) 0.7494
Mother-only employment	0.85 (0.64–1.12) 0.2555	1.02 (0.75–1.37) 0.9185
Dual non-employment	0.82 (0.72–0.93) 0.0028	0.97 (0.83–1.12) 0.6738

Non-adjusted model adjust for: none.

Adjusted model adjust for: child age, child gender, marital status, annual family income, paternal age, maternal age, paternal education level, maternal education level, paternal smoking status, maternal smoking status, paternal alcohol intake status, maternal alcohol intake status, and CES-D scores.

Table 3 shows the associations between paternal employment status and child outcomes, with maternal employment status included as a covariate. In the fully adjusted models, paternal unemployment was significantly associated with higher odds of externalizing problems (adjusted OR = 1.25, 95% CI: 1.03–1.51, *P* = 0.024), whereas paternal not in labor force showed no significant associations with any of the four outcomes.

**Table 3:** Association between paternal employment status and preschoolers' emotional and behavioral problems.

Variables	Non-adjusted model	Adjusted model
	OR (95% CI) *P* value	OR (95% CI) *P* value
Total difficulties		
Employed	1.0	1.0
Not in labor force	1.47 (1.19–1.8) 0.0002	0.99 (0.77–1.25) 0.9028
Unemployed	1.97 (1.65–2.33) 0.0000	1.16 (0.94–1.41) 0.1607
Internalizing problems		
Employed	1.0	1.0
Not in labor force	1.57 (1.29–1.91) 0.0000	1.06 (0.85–1.33) 0.5830
Unemployed	1.87 (1.58–2.21) 0.0000	1.17 (0.96–1.41) 0.1232
Externalizing problems		
Employed	1.0	1.0
Not in labor force	1.21 (0.99–1.48) 0.0547	1.09 (0.87–1.36) 0.4607
Unemployed	1.52 (1.28–1.8) 0.0000	1.25 (1.03–1.51) 0.0237
Prosocial behavior		
Employed	1.0	1.0
Not in labor force	0.94 (0.79–1.12) 0.4815	1.04 (0.86–1.27) 0.6626
Unemployed	0.78 (0.67–0.91) 0.0023	0.93 (0.78–1.11) 0.4050

Non-adjusted model adjust for: none.

Adjusted model adjust for: child age, child gender, marital status, annual family income, paternal age, maternal age, paternal education level, maternal education level, paternal smoking status, maternal smoking status, paternal alcohol intake status, maternal alcohol intake status, maternal employment status and CES-D scores.

Table 4 presents the associations between maternal employment status and child outcomes, with paternal employment status adjusted for. In the fully adjusted models, maternal not in labor force was significantly associated with increased odds of internalizing problems (adjusted OR = 1.11, 95% CI: 1.02–1.21, *P* = 0.019), and maternal unemployment was also associated with higher odds of internalizing problems (adjusted OR = 1.27, 95% CI: 1.02–1.56, *P* = 0.029). Additionally, maternal not in labor force was associated with decreased odds of externalizing problems (adjusted OR = 0.89, 95% CI: 0.82–0.97, *P* = 0.008). No significant associations were found between maternal employment status and total difficulties or prosocial behavior in the adjusted models. Multicollinearity diagnostics indicated no significant collinearity among covariates (all VIFs <2) (Supplementary Table S1). Sensitivity analyses using multiple imputation for missing age data yielded results consistent with the complete-case analyses, supporting the robustness of our findings (Supplementary Table S3).

**Table 4:** Association between maternal employment status and preschoolers' emotional and behavioral problems.

Variables	Non-adjusted model	Adjusted model
	OR (95% CI) *P* value	OR (95% CI) *P* value
Total difficulties		
Employed	1.0	1.0
Not in labor force	1.37 (1.28–1.48) 0.0000	1.01 (0.92–1.1) 0.8757
Unemployed	1.9 (1.57–2.29) 0.0000	1.08 (0.86–1.35) 0.4805
Internalizing problems		
Employed	1.0	1.0
Not in labor force	1.41 (1.32–1.52) 0.0000	1.11 (1.02–1.21) 0.0190
Unemployed	1.99 (1.65–2.38) 0.0000	1.27 (1.02–1.56) 0.0287
Externalizing problems		
Employed	1.0	1.0
Not in labor force	1.01 (0.94–1.08) 0.7484	0.89 (0.82–0.97) 0.0075
Unemployed	1.36 (1.13–1.64) 0.0009	0.97 (0.79–1.2) 0.8061
Prosocial behavior		
Employed	1.0	1.0
Not in labor force	0.91 (0.86–0.97) 0.0026	1.03 (0.96–1.1) 0.4868
Unemployed	0.7 (0.59–0.83) 0.0000	0.84 (0.7–1.02) 0.0722

Non-adjusted model adjust for: none.

Adjusted model adjust for: child age, child gender, marital status, annual family income, paternal age, maternal age, paternal education level, maternal education level, paternal smoking status, maternal smoking status, paternal alcohol intake status, maternal alcohol intake status, paternal employment status and CES-D scores.

### 3.3 Subgroup analysis

Supplementary Table S2 presents the results of stratified analyses examining the association between family employment status and child outcomes across subgroups defined by sociodemographic and parental characteristics. After applying the Benjamini-Hochberg false discovery rate (FDR) correction for multiple testing (with FDR-adjusted *P*-values reported), no statistically significant associations survived the correction across any subgroup or outcome. Although some subgroup-specific estimates showed nominal significance (e.g., dual non-employment vs. dual-earner households among children aged ≥5 years for internalizing problems: OR = 1.52, 95% CI: 1.18–1.94, raw *P* = 0.001; dual non-employment vs. dual-earner households among married parents for internalizing problems: OR = 1.38, 95% CI: 1.14–1.66, raw *P* = 0.001), none remained significant after FDR adjustment (all FDR-adjusted *P*-values >0.05).

## 4 Discussion

This study extends the Family Economic Stress Model to the Chinese context by demonstrating that parental employment status is differentially associated with preschoolers' emotional and behavioral problems in a gender-specific manner. Our findings reveal that paternal unemployment primarily elevates the risk of children's externalizing problems, whereas maternal non-employment—whether unemployment or being out of the labor force—is more strongly linked to internalizing problems. Additionally, at the family level, father-only employment conferred mixed effects (slightly increased internalizing but decreased externalizing problems), while dual non-employment significantly amplified internalizing risk. These results underscore that the effects of parental employment on child adjustment are not uniform but depend on both family employment configuration and parental gender, challenging the common practice of aggregating parental employment status in previous research (17).

Compared with dual-earner households, which served as our reference group and demonstrated the most favorable child outcomes across all domains, families with divergent employment configurations exhibited distinct vulnerability profiles. The association between dual non-employment and elevated internalizing problems aligns closely with the Family Economic Stress Model, which posits that the absence of any parental income generates cumulative economic hardship that manifests in children's emotional distress through disrupted family processes (11, 12). However, our finding that father-only employment was associated with lower externalizing problems, despite slightly elevated internalizing problems, diverges from some Western studies that have typically reported uniformly adverse effects of single-earner father families (15, 24). This discrepancy likely reflects the unique social context of contemporary China, where dual-earner families have become the societal norm and father-only employment may represent a conventional arrangement that, while potentially limiting maternal economic autonomy, preserves paternal breadwinning identity and reduces role-conflict-related family tension.

The independent effect of paternal unemployment on children's externalizing problems represents one of our most salient findings and offers novel insights into the paternal-specific pathway of economic stress. In the Chinese cultural context, fathers are traditionally expected to serve as primary economic providers; unemployment therefore constitutes a profound threat to paternal identity and family stability. Consistent with previous research demonstrating that paternal job loss disrupts parenting quality through increased psychological distress and interparental conflict (12), our results suggest that this disruption manifests primarily in children's conduct problems and aggressive behavior rather than emotional withdrawal. This specificity may arise because paternal distress in the context of unemployment often translates into irritable, inconsistent, or coercive parenting practices that directly model and reinforce externalizing behaviors in preschool-aged children (25). Notably, this pattern remained significant even after adjusting for family income and maternal employment status, suggesting that paternal unemployment carries psychological risks beyond its purely economic implications.

In contrast, maternal non-employment exhibited a distinct association pattern characterized by increased internalizing problems. This finding is particularly noteworthy given the high maternal labor force participation rate in contemporary China and the evolving discourse around maternal employment and child development (15, 24). Our results suggest that maternal unemployment and being out of the labor force may heighten children's emotional symptoms through pathways distinct from those involving paternal unemployment. Mothers who are not employed may experience elevated psychological distress stemming from economic dependence, social isolation, or unfulfilled career aspirations, which can increase children's exposure to anxious or withdrawn emotional modeling (26). Additionally, although non-employed mothers may spend more time with their children, the quality of parent-child interactions may be compromised if maternal mental health is adversely affected (27). The absence of an association between maternal non-employment and externalizing problems, combined with the observed reduction in externalizing risk for children of mothers not in the labor force, further supports the specificity of this pathway, indicating that maternal employment status influences child adjustment primarily through emotional rather than behavioral regulatory mechanisms.

Collectively, these gender-differentiated patterns suggest that the Family Economic Stress Model operates through distinct paternal and maternal pathways in the Chinese context. Paternal employment instability appears to compromise child adjustment primarily via externalizing behavioral pathways, likely mediated by disrupted discipline practices and heightened family conflict. Conversely, maternal employment status influences child outcomes primarily through internalizing emotional pathways, potentially mediated by maternal mental health and the emotional climate of the parent-child relationship. This parental gender specificity has rarely been systematically examined in previous literature, which has often aggregated parental employment or focused exclusively on maternal employment (15, 17). Our findings therefore contribute a more nuanced understanding of how macro-level employment structures interact with culturally embedded gender roles to shape child development. In the context of China's rapid socioeconomic transformation, where traditional gender expectations persist alongside increasing maternal workforce participation, these results highlight the need for family policies that address both paternal economic security and maternal psychosocial well-being.

Subgroup analyses by sociodemographic and parental characteristics revealed no significant effect modification after FDR correction for multiple testing. While this finding precludes the identification of specific high-risk subpopulations, it simultaneously strengthens the generalizability of our core results, suggesting that parental employment influences child mental health through relatively stable mechanisms that transcend variations in child age, gender, family income, and parental education. This robustness is methodologically reassuring and aligns with the universalist perspective of the Family Economic Stress Model, which proposes that economic pressure affects family processes across diverse demographic contexts (11, 12).

These findings carry important implications for policy and intervention. First, employment support programs targeting fathers should recognize that paternal job loss represents a dual risk factor for family economic stability and child behavioral regulation. Interventions might integrate vocational support with parenting skills training to buffer the adverse behavioral consequences of paternal unemployment. Second, maternal employment policies should extend beyond simple labor force participation metrics to address the quality of maternal work and the availability of mental health support for non-employed mothers. Given our finding that maternal non-employment is associated with increased internalizing problems, community-based programs offering social engagement opportunities and psychological support for non-employed mothers may be particularly beneficial. Finally, family-level interventions should adopt a gender-differentiated approach, recognizing that paternal and maternal employment transitions trigger distinct child adjustment challenges requiring tailored preventive strategies.

This study has several limitations that should be acknowledged. First, due to the cross-sectional design, causal inferences cannot be established; our findings should be interpreted as associations rather than causal effects, and the temporal precedence between parental employment status and child outcomes remains unclear. Second, although we refined the classification of parental employment status into four family-level categories and further distinguished between “not in the labor force” and “unemployed” in parent-specific analyses, we were unable to differentiate between voluntary and involuntary non-employment within these groups, nor did we capture job quality dimensions such as work intensity, schedule flexibility, or job satisfaction. These unmeasured aspects may obscure residual heterogeneity in the observed associations. Third, we were unable to formally test mediating pathways—including parental mental health, marital conflict, and grandparental involvement—as specified in the Family Economic Stress Model. Although we controlled for parental depressive symptoms (CES-D) as a covariate in all regression models, other potential mediators were not measured. Fourth, although the SDQ has demonstrated adequate psychometric properties in preschool populations, including children aged 3 years and younger, in previous studies, specific validation studies targeting Chinese 3-year-olds remain relatively limited. Fifth, although we applied Benjamini-Hochberg false discovery rate (FDR) correction to all subgroup analyses to control for Type I error inflation due to multiple testing, these analyses were exploratory in nature; the results should be interpreted cautiously and require further confirmation in future studies with larger sample sizes and pre-specified hypotheses.

## Data Availability

The raw data supporting the conclusions of this article will be made available by the authors, without undue reservation.
