# Supplementary material for: Parental employment status and preschoolers' emotional and behavioral problems in Western China: a cross-sectional study
Source: Front Pediatr. 2026 Aug 19;14:1897604. doi: 10.3389/fped.2026.1897604 (PMC13533897; doi:10.3389/fped.2026.1897604)
Supplement: Supplementary file 1 [file Table1.docx]

**Supplementary Tables**

**Table S1.** Multicollinearity diagnostics for the regression model of preschoolers’ emotional and behavioral problems

| **Variables** | **VIF** |
| --- | --- |
| **Parental employment status** | 1.05 |
| **Child age** | 1.02 |
| **Child gender** | 1.00 |
| **Marital status** | 1.01 |
| **Annual family income** | 1.07 |
| **CES-D** | 1.03 |
| **Paternal age** | 1.66 |
| **Paternal education level** | 1.36 |
| **Paternal smoking status** | 1.18 |
| **Paternal alcohol intake status** | 1.18 |
| **Maternal age** | 1.66 |
| **Maternal education level** | 1.40 |
| **Maternal smoking status** | 1.16 |
| **Maternal alcohol intake status** | 1.12 |
|  |  |

**Table S2. Subgroup analyses of the association between parental employment status and preschoolers’ emotional and behavioral problems**

| **Outcome** | **Stratification variable** | **Stratum** | **Independent variable (ref. Dual-earner households)** | **OR (95% CI)** | **P-value** | **FDR-adjusted P-value** |
| --- | --- | --- | --- | --- | --- | --- |
| **Total difficulties** | **Child age, years** | <4 | **Father-only employment** | 1 (0.81-1.23) | 0.9674 | 0.9878 |
| **Total difficulties** | **Child age, years** | <4 | **Mother-only employment** | 1.06 (0.29-3.11) | 0.9266 | 0.9878 |
| **Total difficulties** | **Child age, years** | <4 | **Dual non-employment** | 1.39 (0.88-2.16) | 0.1516 | 0.5144 |
| **Total difficulties** | **Child age, years** | 4-5 | **Father-only employment** | 1.08 (0.9-1.29) | 0.4104 | 0.7296 |
| **Total difficulties** | **Child age, years** | 4-5 | **Mother-only employment** | 0.81 (0.38-1.59) | 0.5548 | 0.8358 |
| **Total difficulties** | **Child age, years** | 4-5 | **Dual non-employment** | 1.13 (0.79-1.6) | 0.4826 | 0.7726 |
| **Total difficulties** | **Child age, years** | ≥5 | **Father-only employment** | 0.99 (0.86-1.14) | 0.8941 | 0.9721 |
| **Total difficulties** | **Child age, years** | ≥5 | **Mother-only employment** | 1.96 (1.15-3.25) | 0.0112 | 0.1764 |
| **Total difficulties** | **Child age, years** | ≥5 | **Dual non-employment** | 1.21 (0.92-1.57) | 0.1630 | 0.5209 |
| **Total difficulties** | **Child gender** | Boys | **Father-only employment** | 1.05 (0.91-1.2) | 0.5141 | 0.8025 |
| **Total difficulties** | **Child gender** | Boys | **Mother-only employment** | 1.42 (0.84-2.32) | 0.1715 | 0.5309 |
| **Total difficulties** | **Child gender** | Boys | **Dual non-employment** | 1.13 (0.87-1.46) | 0.3576 | 0.7108 |
| **Total difficulties** | **Child gender** | Girls | **Father-only employment** | 0.99 (0.86-1.14) | 0.8918 | 0.9721 |
| **Total difficulties** | **Child gender** | Girls | **Mother-only employment** | 1.23 (0.65-2.21) | 0.5066 | 0.7973 |
| **Total difficulties** | **Child gender** | Girls | **Dual non-employment** | 1.29 (0.98-1.7) | 0.0697 | 0.3995 |
| **Total difficulties** | **Marital status** | Married/Living with partner | **Father-only employment** | 1.01 (0.91-1.11) | 0.8821 | 0.9706 |
| **Total difficulties** | **Marital status** | Married/Living with partner | **Mother-only employment** | 1.27 (0.83-1.9) | 0.2599 | 0.6199 |
| **Total difficulties** | **Marital status** | Married/Living with partner | **Dual non-employment** | 1.23 (1.01-1.49) | 0.0407 | 0.3126 |
| **Total difficulties** | **Marital status** | Others | **Father-only employment** | 1.67 (0.86-3.21) | 0.1279 | 0.5144 |
| **Total difficulties** | **Marital status** | Others | **Mother-only employment** | 2.33 (0.65-7.87) | 0.1797 | 0.5309 |
| **Total difficulties** | **Marital status** | Others | **Dual non-employment** | 1.05 (0.45-2.38) | 0.9156 | 0.9848 |
| **Total difficulties** | **Annual family income, in thousands, RMB** | < 30 | **Father-only employment** | 1.09 (0.95-1.25) | 0.2204 | 0.5776 |
| **Total difficulties** | **Annual family income, in thousands, RMB** | < 30 | **Mother-only employment** | 1.12 (0.69-1.77) | 0.6431 | 0.8588 |
| **Total difficulties** | **Annual family income, in thousands, RMB** | < 30 | **Dual non-employment** | 1.16 (0.93-1.44) | 0.1942 | 0.5443 |
| **Total difficulties** | **Annual family income, in thousands, RMB** | ≥ 30 to 100 | **Father-only employment** | 0.93 (0.79-1.09) | 0.3658 | 0.7108 |
| **Total difficulties** | **Annual family income, in thousands, RMB** | ≥ 30 to 100 | **Mother-only employment** | 2 (0.95-3.93) | 0.0535 | 0.3482 |
| **Total difficulties** | **Annual family income, in thousands, RMB** | ≥ 30 to 100 | **Dual non-employment** | 1.44 (0.95-2.14) | 0.0790 | 0.4030 |
| **Total difficulties** | **Annual family income, in thousands, RMB** | ≥ 100 | **Father-only employment** | 1 (0.71-1.39) | 0.9780 | 0.9886 |
| **Total difficulties** | **Annual family income, in thousands, RMB** | ≥ 100 | **Mother-only employment** | 3.01 (0.15-24.45) | 0.3478 | 0.7066 |
| **Total difficulties** | **Annual family income, in thousands, RMB** | ≥ 100 | **Dual non-employment** | 2.39 (0.67-7.45) | 0.1488 | 0.5144 |
| **Total difficulties** | **Depression risk** | No | **Father-only employment** | 1 (0.89-1.12) | 0.9999 | 0.9999 |
| **Total difficulties** | **Depression risk** | No | **Mother-only employment** | 1.51 (0.91-2.4) | 0.0918 | 0.4406 |
| **Total difficulties** | **Depression risk** | No | **Dual non-employment** | 1.17 (0.93-1.48) | 0.1788 | 0.5309 |
| **Total difficulties** | **Depression risk** | Yes | **Father-only employment** | 1.09 (0.9-1.32) | 0.3930 | 0.7216 |
| **Total difficulties** | **Depression risk** | Yes | **Mother-only employment** | 1.14 (0.6-2.12) | 0.6774 | 0.8820 |
| **Total difficulties** | **Depression risk** | Yes | **Dual non-employment** | 1.35 (0.97-1.87) | 0.0763 | 0.4030 |
| **Total difficulties** | **Paternal age, years** | <30 | **Father-only employment** | 0.95 (0.68-1.31) | 0.7412 | 0.9036 |
| **Total difficulties** | **Paternal age, years** | <30 | **Mother-only employment** | 0.87 (0.18-3.22) | 0.8412 | 0.9606 |
| **Total difficulties** | **Paternal age, years** | <30 | **Dual non-employment** | 1.73 (0.79-3.68) | 0.1614 | 0.5209 |
| **Total difficulties** | **Paternal age, years** | 30-44 | **Father-only employment** | 1.02 (0.92-1.13) | 0.7246 | 0.9036 |
| **Total difficulties** | **Paternal age, years** | 30-44 | **Mother-only employment** | 1.39 (0.89-2.11) | 0.1338 | 0.5144 |
| **Total difficulties** | **Paternal age, years** | 30-44 | **Dual non-employment** | 1.18 (0.96-1.45) | 0.1141 | 0.4923 |
| **Total difficulties** | **Paternal age, years** | ≥45 | **Father-only employment** | 1.26 (0.74-2.14) | 0.3899 | 0.7216 |
| **Total difficulties** | **Paternal age, years** | ≥45 | **Mother-only employment** | 1.58 (0.44-5.06) | 0.4578 | 0.7577 |
| **Total difficulties** | **Paternal age, years** | ≥45 | **Dual non-employment** | 1.23 (0.57-2.57) | 0.5903 | 0.8458 |
| **Total difficulties** | **Paternal education level** | ≤ Junior high school | **Father-only employment** | 1.06 (0.92-1.23) | 0.4010 | 0.7216 |
| **Total difficulties** | **Paternal education level** | ≤ Junior high school | **Mother-only employment** | 1.19 (0.68-2.01) | 0.5364 | 0.8339 |
| **Total difficulties** | **Paternal education level** | ≤ Junior high school | **Dual non-employment** | 1.09 (0.86-1.38) | 0.4657 | 0.7642 |
| **Total difficulties** | **Paternal education level** | High school diploma and junior college | **Father-only employment** | 0.96 (0.82-1.11) | 0.5638 | 0.8379 |
| **Total difficulties** | **Paternal education level** | High school diploma and junior college | **Mother-only employment** | 1.78 (0.98-3.12) | 0.0499 | 0.3422 |
| **Total difficulties** | **Paternal education level** | High school diploma and junior college | **Dual non-employment** | 1.65 (1.14-2.34) | 0.0062 | 0.1323 |
| **Total difficulties** | **Paternal education level** | ≥ Undergraduate degree | **Father-only employment** | 1.05 (0.73-1.48) | 0.7857 | 0.9341 |
| **Total difficulties** | **Paternal education level** | ≥ Undergraduate degree | **Mother-only employment** | 0.39 (0.02-2.39) | 0.3948 | 0.7216 |
| **Total difficulties** | **Paternal education level** | ≥ Undergraduate degree | **Dual non-employment** | 1.86 (0.5-5.69) | 0.3055 | 0.6488 |
| **Total difficulties** | **Paternal smoking status** | No | **Father-only employment** | 1.02 (0.9-1.17) | 0.7375 | 0.9036 |
| **Total difficulties** | **Paternal smoking status** | No | **Mother-only employment** | 1.44 (0.84-2.37) | 0.1679 | 0.5242 |
| **Total difficulties** | **Paternal smoking status** | No | **Dual non-employment** | 1.42 (1.11-1.8) | 0.0044 | 0.1234 |
| **Total difficulties** | **Paternal smoking status** | Yes | **Father-only employment** | 1.01 (0.87-1.18) | 0.8610 | 0.9696 |
| **Total difficulties** | **Paternal smoking status** | Yes | **Mother-only employment** | 1.19 (0.64-2.11) | 0.5668 | 0.8379 |
| **Total difficulties** | **Paternal smoking status** | Yes | **Dual non-employment** | 0.94 (0.68-1.28) | 0.7066 | 0.8936 |
| **Total difficulties** | **Paternal alcohol intake status** | No | **Father-only employment** | 1.05 (0.93-1.18) | 0.4311 | 0.7408 |
| **Total difficulties** | **Paternal alcohol intake status** | No | **Mother-only employment** | 1.69 (1.06-2.64) | 0.0247 | 0.2313 |
| **Total difficulties** | **Paternal alcohol intake status** | No | **Dual non-employment** | 1.18 (0.94-1.49) | 0.1526 | 0.5144 |
| **Total difficulties** | **Paternal alcohol intake status** | Yes | **Father-only employment** | 0.96 (0.8-1.15) | 0.6463 | 0.8588 |
| **Total difficulties** | **Paternal alcohol intake status** | Yes | **Mother-only employment** | 0.82 (0.37-1.67) | 0.5996 | 0.8468 |
| **Total difficulties** | **Paternal alcohol intake status** | Yes | **Dual non-employment** | 1.28 (0.91-1.78) | 0.1458 | 0.5144 |
| **Total difficulties** | **Maternal age, years** | <30 | **Father-only employment** | 1.01 (0.81-1.26) | 0.9432 | 0.9878 |
| **Total difficulties** | **Maternal age, years** | <30 | **Mother-only employment** | 2.44 (0.87-6.54) | 0.0804 | 0.4030 |
| **Total difficulties** | **Maternal age, years** | <30 | **Dual non-employment** | 1.97 (1.23-3.13) | 0.0045 | 0.1234 |
| **Total difficulties** | **Maternal age, years** | 30-44 | **Father-only employment** | 1.02 (0.91-1.14) | 0.7717 | 0.9303 |
| **Total difficulties** | **Maternal age, years** | 30-44 | **Mother-only employment** | 1.19 (0.76-1.82) | 0.4334 | 0.7408 |
| **Total difficulties** | **Maternal age, years** | 30-44 | **Dual non-employment** | 1.12 (0.91-1.39) | 0.2814 | 0.6261 |
| **Total difficulties** | **Maternal age, years** | ≥45 | **Father-only employment** | 1.17 (0.43-3.21) | 0.7522 | 0.9112 |
| **Total difficulties** | **Maternal age, years** | ≥45 | **Mother-only employment** | 1.12 (0.12-7.61) | 0.9126 | 0.9848 |
| **Total difficulties** | **Maternal age, years** | ≥45 | **Dual non-employment** | 0.41 (0.09-1.65) | 0.2226 | 0.5776 |
| **Total difficulties** | **Maternal education level** | ≤ Junior high school | **Father-only employment** | 1.03 (0.89-1.2) | 0.6875 | 0.8841 |
| **Total difficulties** | **Maternal education level** | ≤ Junior high school | **Mother-only employment** | 1.16 (0.53-2.41) | 0.6907 | 0.8841 |
| **Total difficulties** | **Maternal education level** | ≤ Junior high school | **Dual non-employment** | 1.12 (0.88-1.42) | 0.3500 | 0.7074 |
| **Total difficulties** | **Maternal education level** | High school diploma and junior college | **Father-only employment** | 1.02 (0.88-1.17) | 0.8034 | 0.9406 |
| **Total difficulties** | **Maternal education level** | High school diploma and junior college | **Mother-only employment** | 1.53 (0.88-2.58) | 0.1206 | 0.4980 |
| **Total difficulties** | **Maternal education level** | High school diploma and junior college | **Dual non-employment** | 1.38 (0.98-1.93) | 0.0623 | 0.3738 |
| **Total difficulties** | **Maternal education level** | ≥ Undergraduate degree | **Father-only employment** | 0.83 (0.55-1.22) | 0.3621 | 0.7108 |
| **Total difficulties** | **Maternal education level** | ≥ Undergraduate degree | **Mother-only employment** | 1.12 (0.45-2.5) | 0.7980 | 0.9390 |
| **Total difficulties** | **Maternal education level** | ≥ Undergraduate degree | **Dual non-employment** | 4.06 (1.34-11.27) | 0.0085 | 0.1484 |
| **Total difficulties** | **Maternal smoking status** | No | **Father-only employment** | 1.02 (0.92-1.13) | 0.7354 | 0.9036 |
| **Total difficulties** | **Maternal smoking status** | No | **Mother-only employment** | 1.37 (0.91-2.03) | 0.1173 | 0.4950 |
| **Total difficulties** | **Maternal smoking status** | No | **Dual non-employment** | 1.23 (1.01-1.49) | 0.0366 | 0.2976 |
| **Total difficulties** | **Maternal smoking status** | Yes | **Father-only employment** | 1.13 (0.67-1.89) | 0.6440 | 0.8588 |
| **Total difficulties** | **Maternal smoking status** | Yes | **Mother-only employment** | 0.97 (0.13-4.62) | 0.9698 | 0.9878 |
| **Total difficulties** | **Maternal smoking status** | Yes | **Dual non-employment** | 0.79 (0.23-2.36) | 0.6875 | 0.8841 |
| **Total difficulties** | **Maternal alcohol intake status** | No | **Father-only employment** | 1.01 (0.91-1.12) | 0.7996 | 0.9390 |
| **Total difficulties** | **Maternal alcohol intake status** | No | **Mother-only employment** | 1.34 (0.89-1.98) | 0.1470 | 0.5144 |
| **Total difficulties** | **Maternal alcohol intake status** | No | **Dual non-employment** | 1.22 (1-1.47) | 0.0457 | 0.3340 |
| **Total difficulties** | **Maternal alcohol intake status** | Yes | **Father-only employment** | 1.15 (0.72-1.82) | 0.5572 | 0.8358 |
| **Total difficulties** | **Maternal alcohol intake status** | Yes | **Mother-only employment** | 1.2 (0.15-7.05) | 0.8438 | 0.9606 |
| **Total difficulties** | **Maternal alcohol intake status** | Yes | **Dual non-employment** | 1 (0.31-3) | 0.9940 | 0.9992 |
| **Internalizing problems** | **Child age, years** | <4 | **Father-only employment** | 1.24 (1.01-1.53) | 0.0398 | 0.3119 |
| **Internalizing problems** | **Child age, years** | <4 | **Mother-only employment** | 0.22 (0.01-1.12) | 0.1472 | 0.5144 |
| **Internalizing problems** | **Child age, years** | <4 | **Dual non-employment** | 1.37 (0.86-2.14) | 0.1784 | 0.5309 |
| **Internalizing problems** | **Child age, years** | 4-5 | **Father-only employment** | 1.1 (0.93-1.3) | 0.2679 | 0.6235 |
| **Internalizing problems** | **Child age, years** | 4-5 | **Mother-only employment** | 0.65 (0.3-1.28) | 0.2402 | 0.5989 |
| **Internalizing problems** | **Child age, years** | 4-5 | **Dual non-employment** | 1.21 (0.86-1.68) | 0.2723 | 0.6261 |
| **Internalizing problems** | **Child age, years** | ≥5 | **Father-only employment** | 1.11 (0.97-1.28) | 0.1203 | 0.4980 |
| **Internalizing problems** | **Child age, years** | ≥5 | **Mother-only employment** | 2.05 (1.22-3.36) | 0.0053 | 0.1288 |
| **Internalizing problems** | **Child age, years** | ≥5 | **Dual non-employment** | 1.52 (1.18-1.94) | 0.0009 | 0.0494 |
| **Internalizing problems** | **Child gender** | Boys | **Father-only employment** | 1.08 (0.94-1.23) | 0.2863 | 0.6282 |
| **Internalizing problems** | **Child gender** | Boys | **Mother-only employment** | 1.33 (0.79-2.18) | 0.2671 | 0.6235 |
| **Internalizing problems** | **Child gender** | Boys | **Dual non-employment** | 1.38 (1.07-1.76) | 0.0124 | 0.1764 |
| **Internalizing problems** | **Child gender** | Girls | **Father-only employment** | 1.19 (1.04-1.36) | 0.0095 | 0.1586 |
| **Internalizing problems** | **Child gender** | Girls | **Mother-only employment** | 0.95 (0.5-1.71) | 0.8694 | 0.9705 |
| **Internalizing problems** | **Child gender** | Girls | **Dual non-employment** | 1.39 (1.07-1.81) | 0.0143 | 0.1843 |
| **Internalizing problems** | **Marital status** | Married/Living with partner | **Father-only employment** | 1.12 (1.02-1.23) | 0.0194 | 0.2117 |
| **Internalizing problems** | **Marital status** | Married/Living with partner | **Mother-only employment** | 1.13 (0.74-1.68) | 0.5735 | 0.8397 |
| **Internalizing problems** | **Marital status** | Married/Living with partner | **Dual non-employment** | 1.38 (1.14-1.66) | 0.0008 | 0.0494 |
| **Internalizing problems** | **Marital status** | Others | **Father-only employment** | 1.62 (0.86-3.02) | 0.1318 | 0.5144 |
| **Internalizing problems** | **Marital status** | Others | **Mother-only employment** | 1.72 (0.46-5.78) | 0.3977 | 0.7216 |
| **Internalizing problems** | **Marital status** | Others | **Dual non-employment** | 1.49 (0.68-3.27) | 0.3154 | 0.6618 |
| **Internalizing problems** | **Annual family income, in thousands, RMB** | < 30 | **Father-only employment** | 1.13 (0.99-1.28) | 0.0695 | 0.3995 |
| **Internalizing problems** | **Annual family income, in thousands, RMB** | < 30 | **Mother-only employment** | 0.95 (0.58-1.5) | 0.8248 | 0.9533 |
| **Internalizing problems** | **Annual family income, in thousands, RMB** | < 30 | **Dual non-employment** | 1.34 (1.08-1.65) | 0.0072 | 0.1445 |
| **Internalizing problems** | **Annual family income, in thousands, RMB** | ≥ 30 to 100 | **Father-only employment** | 1.14 (0.98-1.33) | 0.0939 | 0.4416 |
| **Internalizing problems** | **Annual family income, in thousands, RMB** | ≥ 30 to 100 | **Mother-only employment** | 1.9 (0.91-3.73) | 0.0713 | 0.4013 |
| **Internalizing problems** | **Annual family income, in thousands, RMB** | ≥ 30 to 100 | **Dual non-employment** | 1.39 (0.92-2.07) | 0.1054 | 0.4706 |
| **Internalizing problems** | **Annual family income, in thousands, RMB** | ≥ 100 | **Father-only employment** | 1.13 (0.82-1.54) | 0.4425 | 0.7428 |
| **Internalizing problems** | **Annual family income, in thousands, RMB** | ≥ 100 | **Mother-only employment** | 0 (NA-132163949.75) | 0.9652 | 0.9878 |
| **Internalizing problems** | **Annual family income, in thousands, RMB** | ≥ 100 | **Dual non-employment** | 2.61 (0.76-7.85) | 0.0992 | 0.4482 |
| **Internalizing problems** | **Depression risk** | No | **Father-only employment** | 1.1 (0.98-1.22) | 0.0943 | 0.4416 |
| **Internalizing problems** | **Depression risk** | No | **Mother-only employment** | 1.25 (0.75-2) | 0.3665 | 0.7108 |
| **Internalizing problems** | **Depression risk** | No | **Dual non-employment** | 1.38 (1.11-1.71) | 0.0038 | 0.1234 |
| **Internalizing problems** | **Depression risk** | Yes | **Father-only employment** | 1.26 (1.04-1.53) | 0.0190 | 0.2117 |
| **Internalizing problems** | **Depression risk** | Yes | **Mother-only employment** | 1.01 (0.53-1.89) | 0.9659 | 0.9878 |
| **Internalizing problems** | **Depression risk** | Yes | **Dual non-employment** | 1.48 (1.06-2.06) | 0.0194 | 0.2117 |
| **Internalizing problems** | **Paternal age, years** | <30 | **Father-only employment** | 1.3 (0.95-1.76) | 0.0959 | 0.4437 |
| **Internalizing problems** | **Paternal age, years** | <30 | **Mother-only employment** | 0.22 (0.01-1.19) | 0.1527 | 0.5144 |
| **Internalizing problems** | **Paternal age, years** | <30 | **Dual non-employment** | 1.89 (0.87-3.98) | 0.0975 | 0.4457 |
| **Internalizing problems** | **Paternal age, years** | 30-44 | **Father-only employment** | 1.11 (1-1.22) | 0.0491 | 0.3422 |
| **Internalizing problems** | **Paternal age, years** | 30-44 | **Mother-only employment** | 1.34 (0.87-2.02) | 0.1738 | 0.5309 |
| **Internalizing problems** | **Paternal age, years** | 30-44 | **Dual non-employment** | 1.35 (1.11-1.64) | 0.0024 | 0.0922 |
| **Internalizing problems** | **Paternal age, years** | ≥45 | **Father-only employment** | 1.31 (0.79-2.18) | 0.2946 | 0.6355 |
| **Internalizing problems** | **Paternal age, years** | ≥45 | **Mother-only employment** | 0.9 (0.22-3) | 0.8659 | 0.9705 |
| **Internalizing problems** | **Paternal age, years** | ≥45 | **Dual non-employment** | 1.34 (0.65-2.71) | 0.4276 | 0.7408 |
| **Internalizing problems** | **Paternal education level** | ≤ Junior high school | **Father-only employment** | 1.03 (0.9-1.19) | 0.6409 | 0.8588 |
| **Internalizing problems** | **Paternal education level** | ≤ Junior high school | **Mother-only employment** | 0.99 (0.56-1.67) | 0.9668 | 0.9878 |
| **Internalizing problems** | **Paternal education level** | ≤ Junior high school | **Dual non-employment** | 1.14 (0.91-1.43) | 0.2395 | 0.5989 |
| **Internalizing problems** | **Paternal education level** | High school diploma and junior college | **Father-only employment** | 1.17 (1.02-1.35) | 0.0235 | 0.2256 |
| **Internalizing problems** | **Paternal education level** | High school diploma and junior college | **Mother-only employment** | 1.32 (0.71-2.34) | 0.3592 | 0.7108 |
| **Internalizing problems** | **Paternal education level** | High school diploma and junior college | **Dual non-employment** | 2.03 (1.44-2.83) | 0.0000 | 0.0000 |
| **Internalizing problems** | **Paternal education level** | ≥ Undergraduate degree | **Father-only employment** | 1.32 (0.97-1.79) | 0.0738 | 0.4030 |
| **Internalizing problems** | **Paternal education level** | ≥ Undergraduate degree | **Mother-only employment** | 1 (0.14-4.39) | 0.9981 | 0.9999 |
| **Internalizing problems** | **Paternal education level** | ≥ Undergraduate degree | **Dual non-employment** | 1.42 (0.38-4.27) | 0.5585 | 0.8358 |
| **Internalizing problems** | **Paternal smoking status** | No | **Father-only employment** | 1.1 (0.97-1.25) | 0.1355 | 0.5144 |
| **Internalizing problems** | **Paternal smoking status** | No | **Mother-only employment** | 0.78 (0.43-1.35) | 0.4004 | 0.7216 |
| **Internalizing problems** | **Paternal smoking status** | No | **Dual non-employment** | 1.68 (1.34-2.12) | 0.0000 | 0.0000 |
| **Internalizing problems** | **Paternal smoking status** | Yes | **Father-only employment** | 1.18 (1.02-1.36) | 0.0257 | 0.2350 |
| **Internalizing problems** | **Paternal smoking status** | Yes | **Mother-only employment** | 1.72 (0.98-2.95) | 0.0512 | 0.3449 |
| **Internalizing problems** | **Paternal smoking status** | Yes | **Dual non-employment** | 1.01 (0.74-1.36) | 0.9426 | 0.9878 |
| **Internalizing problems** | **Paternal alcohol intake status** | No | **Father-only employment** | 1.11 (0.99-1.24) | 0.0817 | 0.4030 |
| **Internalizing problems** | **Paternal alcohol intake status** | No | **Mother-only employment** | 1.07 (0.65-1.71) | 0.7740 | 0.9303 |
| **Internalizing problems** | **Paternal alcohol intake status** | No | **Dual non-employment** | 1.36 (1.09-1.69) | 0.0056 | 0.1288 |
| **Internalizing problems** | **Paternal alcohol intake status** | Yes | **Father-only employment** | 1.21 (1.02-1.43) | 0.0296 | 0.2577 |
| **Internalizing problems** | **Paternal alcohol intake status** | Yes | **Mother-only employment** | 1.37 (0.68-2.61) | 0.3528 | 0.7093 |
| **Internalizing problems** | **Paternal alcohol intake status** | Yes | **Dual non-employment** | 1.47 (1.05-2.03) | 0.0210 | 0.2122 |
| **Internalizing problems** | **Maternal age, years** | <30 | **Father-only employment** | 1.24 (1-1.53) | 0.0461 | 0.3340 |
| **Internalizing problems** | **Maternal age, years** | <30 | **Mother-only employment** | 1.52 (0.5-4.15) | 0.4295 | 0.7408 |
| **Internalizing problems** | **Maternal age, years** | <30 | **Dual non-employment** | 2.15 (1.34-3.4) | 0.0012 | 0.0555 |
| **Internalizing problems** | **Maternal age, years** | 30-44 | **Father-only employment** | 1.1 (0.99-1.22) | 0.0721 | 0.4013 |
| **Internalizing problems** | **Maternal age, years** | 30-44 | **Mother-only employment** | 1.11 (0.71-1.69) | 0.6302 | 0.8588 |
| **Internalizing problems** | **Maternal age, years** | 30-44 | **Dual non-employment** | 1.29 (1.05-1.57) | 0.0143 | 0.1843 |
| **Internalizing problems** | **Maternal age, years** | ≥45 | **Father-only employment** | 1.14 (0.43-3.01) | 0.7953 | 0.9390 |
| **Internalizing problems** | **Maternal age, years** | ≥45 | **Mother-only employment** | 0.66 (0.03-5.6) | 0.7396 | 0.9036 |
| **Internalizing problems** | **Maternal age, years** | ≥45 | **Dual non-employment** | 0.77 (0.2-2.76) | 0.6930 | 0.8841 |
| **Internalizing problems** | **Maternal education level** | ≤ Junior high school | **Father-only employment** | 1.1 (0.95-1.28) | 0.1900 | 0.5432 |
| **Internalizing problems** | **Maternal education level** | ≤ Junior high school | **Mother-only employment** | 1.09 (0.5-2.25) | 0.8138 | 0.9470 |
| **Internalizing problems** | **Maternal education level** | ≤ Junior high school | **Dual non-employment** | 1.21 (0.96-1.52) | 0.1106 | 0.4882 |
| **Internalizing problems** | **Maternal education level** | High school diploma and junior college | **Father-only employment** | 1.14 (1-1.31) | 0.0460 | 0.3340 |
| **Internalizing problems** | **Maternal education level** | High school diploma and junior college | **Mother-only employment** | 1.39 (0.8-2.33) | 0.2218 | 0.5776 |
| **Internalizing problems** | **Maternal education level** | High school diploma and junior college | **Dual non-employment** | 1.69 (1.22-2.32) | 0.0013 | 0.0555 |
| **Internalizing problems** | **Maternal education level** | ≥ Undergraduate degree | **Father-only employment** | 1.08 (0.76-1.52) | 0.6548 | 0.8670 |
| **Internalizing problems** | **Maternal education level** | ≥ Undergraduate degree | **Mother-only employment** | 0.72 (0.26-1.7) | 0.4883 | 0.7748 |
| **Internalizing problems** | **Maternal education level** | ≥ Undergraduate degree | **Dual non-employment** | 4.21 (1.46-11.66) | 0.0057 | 0.1288 |
| **Internalizing problems** | **Maternal smoking status** | No | **Father-only employment** | 1.14 (1.04-1.25) | 0.0077 | 0.1445 |
| **Internalizing problems** | **Maternal smoking status** | No | **Mother-only employment** | 1.23 (0.82-1.81) | 0.3117 | 0.6577 |
| **Internalizing problems** | **Maternal smoking status** | No | **Dual non-employment** | 1.42 (1.18-1.71) | 0.0002 | 0.0192 |
| **Internalizing problems** | **Maternal smoking status** | Yes | **Father-only employment** | 0.99 (0.59-1.63) | 0.9676 | 0.9878 |
| **Internalizing problems** | **Maternal smoking status** | Yes | **Mother-only employment** | 0.33 (0.02-2.04) | 0.3171 | 0.6618 |
| **Internalizing problems** | **Maternal smoking status** | Yes | **Dual non-employment** | 0.67 (0.2-1.92) | 0.4785 | 0.7720 |
| **Internalizing problems** | **Maternal alcohol intake status** | No | **Father-only employment** | 1.13 (1.03-1.25) | 0.0120 | 0.1764 |
| **Internalizing problems** | **Maternal alcohol intake status** | No | **Mother-only employment** | 1.19 (0.79-1.76) | 0.3807 | 0.7216 |
| **Internalizing problems** | **Maternal alcohol intake status** | No | **Dual non-employment** | 1.41 (1.18-1.7) | 0.0002 | 0.0192 |
| **Internalizing problems** | **Maternal alcohol intake status** | Yes | **Father-only employment** | 1.2 (0.76-1.86) | 0.4342 | 0.7408 |
| **Internalizing problems** | **Maternal alcohol intake status** | Yes | **Mother-only employment** | 0.44 (0.02-3.12) | 0.4745 | 0.7688 |
| **Internalizing problems** | **Maternal alcohol intake status** | Yes | **Dual non-employment** | 0.8 (0.25-2.3) | 0.6887 | 0.8841 |
| **Externalizing problems** | **Child age, years** | <4 | **Father-only employment** | 0.79 (0.65-0.96) | 0.0177 | 0.2117 |
| **Externalizing problems** | **Child age, years** | <4 | **Mother-only employment** | 1.74 (0.66-4.43) | 0.2474 | 0.6090 |
| **Externalizing problems** | **Child age, years** | <4 | **Dual non-employment** | 1.06 (0.69-1.62) | 0.7777 | 0.9303 |
| **Externalizing problems** | **Child age, years** | 4-5 | **Father-only employment** | 0.94 (0.8-1.1) | 0.4360 | 0.7408 |
| **Externalizing problems** | **Child age, years** | 4-5 | **Mother-only employment** | 0.93 (0.48-1.68) | 0.8078 | 0.9428 |
| **Externalizing problems** | **Child age, years** | 4-5 | **Dual non-employment** | 1.1 (0.79-1.52) | 0.5594 | 0.8358 |
| **Externalizing problems** | **Child age, years** | ≥5 | **Father-only employment** | 0.94 (0.82-1.07) | 0.3630 | 0.7108 |
| **Externalizing problems** | **Child age, years** | ≥5 | **Mother-only employment** | 1.41 (0.83-2.31) | 0.1867 | 0.5390 |
| **Externalizing problems** | **Child age, years** | ≥5 | **Dual non-employment** | 1.16 (0.9-1.48) | 0.2386 | 0.5989 |
| **Externalizing problems** | **Child gender** | Boys | **Father-only employment** | 0.88 (0.78-1) | 0.0490 | 0.3422 |
| **Externalizing problems** | **Child gender** | Boys | **Mother-only employment** | 1.57 (0.98-2.45) | 0.0533 | 0.3482 |
| **Externalizing problems** | **Child gender** | Boys | **Dual non-employment** | 1.01 (0.79-1.29) | 0.9136 | 0.9848 |
| **Externalizing problems** | **Child gender** | Girls | **Father-only employment** | 0.93 (0.81-1.06) | 0.2677 | 0.6235 |
| **Externalizing problems** | **Child gender** | Girls | **Mother-only employment** | 0.87 (0.46-1.54) | 0.6386 | 0.8588 |
| **Externalizing problems** | **Child gender** | Girls | **Dual non-employment** | 1.23 (0.94-1.59) | 0.1227 | 0.5012 |
| **Externalizing problems** | **Marital status** | Married/Living with partner | **Father-only employment** | 0.9 (0.82-0.99) | 0.0235 | 0.2256 |
| **Externalizing problems** | **Marital status** | Married/Living with partner | **Mother-only employment** | 1.19 (0.81-1.73) | 0.3707 | 0.7153 |
| **Externalizing problems** | **Marital status** | Married/Living with partner | **Dual non-employment** | 1.13 (0.93-1.35) | 0.2075 | 0.5651 |
| **Externalizing problems** | **Marital status** | Others | **Father-only employment** | 1.35 (0.69-2.6) | 0.3792 | 0.7216 |
| **Externalizing problems** | **Marital status** | Others | **Mother-only employment** | 2.37 (0.69-7.7) | 0.1549 | 0.5172 |
| **Externalizing problems** | **Marital status** | Others | **Dual non-employment** | 1.22 (0.53-2.73) | 0.6331 | 0.8588 |
| **Externalizing problems** | **Annual family income, in thousands, RMB** | < 30 | **Father-only employment** | 0.88 (0.77-0.99) | 0.0372 | 0.2976 |
| **Externalizing problems** | **Annual family income, in thousands, RMB** | < 30 | **Mother-only employment** | 1.19 (0.77-1.81) | 0.4283 | 0.7408 |
| **Externalizing problems** | **Annual family income, in thousands, RMB** | < 30 | **Dual non-employment** | 1 (0.81-1.23) | 0.9825 | 0.9902 |
| **Externalizing problems** | **Annual family income, in thousands, RMB** | ≥ 30 to 100 | **Father-only employment** | 0.84 (0.72-0.97) | 0.0204 | 0.2117 |
| **Externalizing problems** | **Annual family income, in thousands, RMB** | ≥ 30 to 100 | **Mother-only employment** | 1.44 (0.71-2.77) | 0.2837 | 0.6261 |
| **Externalizing problems** | **Annual family income, in thousands, RMB** | ≥ 30 to 100 | **Dual non-employment** | 1.32 (0.89-1.93) | 0.1526 | 0.5144 |
| **Externalizing problems** | **Annual family income, in thousands, RMB** | ≥ 100 | **Father-only employment** | 1.35 (1.03-1.76) | 0.0302 | 0.2577 |
| **Externalizing problems** | **Annual family income, in thousands, RMB** | ≥ 100 | **Mother-only employment** | 0 (NA-123644747.74) | 0.9642 | 0.9878 |
| **Externalizing problems** | **Annual family income, in thousands, RMB** | ≥ 100 | **Dual non-employment** | 4.27 (1.45-12.82) | 0.0079 | 0.1445 |
| **Externalizing problems** | **Depression risk** | No | **Father-only employment** | 0.92 (0.83-1.02) | 0.1135 | 0.4923 |
| **Externalizing problems** | **Depression risk** | No | **Mother-only employment** | 1.3 (0.82-1.99) | 0.2446 | 0.6060 |
| **Externalizing problems** | **Depression risk** | No | **Dual non-employment** | 1.02 (0.82-1.27) | 0.8455 | 0.9606 |
| **Externalizing problems** | **Depression risk** | Yes | **Father-only employment** | 0.86 (0.71-1.05) | 0.1374 | 0.5144 |
| **Externalizing problems** | **Depression risk** | Yes | **Mother-only employment** | 1.18 (0.63-2.18) | 0.5972 | 0.8468 |
| **Externalizing problems** | **Depression risk** | Yes | **Dual non-employment** | 1.37 (0.99-1.91) | 0.0581 | 0.3676 |
| **Externalizing problems** | **Paternal age, years** | <30 | **Father-only employment** | 0.79 (0.59-1.07) | 0.1342 | 0.5144 |
| **Externalizing problems** | **Paternal age, years** | <30 | **Mother-only employment** | 0.7 (0.15-2.48) | 0.6042 | 0.8468 |
| **Externalizing problems** | **Paternal age, years** | <30 | **Dual non-employment** | 1.47 (0.69-3.03) | 0.3058 | 0.6488 |
| **Externalizing problems** | **Paternal age, years** | 30-44 | **Father-only employment** | 0.91 (0.83-1) | 0.0608 | 0.3730 |
| **Externalizing problems** | **Paternal age, years** | 30-44 | **Mother-only employment** | 1.34 (0.89-1.97) | 0.1515 | 0.5144 |
| **Externalizing problems** | **Paternal age, years** | 30-44 | **Dual non-employment** | 1.09 (0.9-1.33) | 0.3651 | 0.7108 |
| **Externalizing problems** | **Paternal age, years** | ≥45 | **Father-only employment** | 0.99 (0.62-1.56) | 0.9511 | 0.9878 |
| **Externalizing problems** | **Paternal age, years** | ≥45 | **Mother-only employment** | 1.25 (0.36-3.86) | 0.7074 | 0.8936 |
| **Externalizing problems** | **Paternal age, years** | ≥45 | **Dual non-employment** | 1.12 (0.57-2.16) | 0.7335 | 0.9036 |
| **Externalizing problems** | **Paternal education level** | ≤ Junior high school | **Father-only employment** | 1.01 (0.88-1.16) | 0.8938 | 0.9721 |
| **Externalizing problems** | **Paternal education level** | ≤ Junior high school | **Mother-only employment** | 1.21 (0.72-1.99) | 0.4599 | 0.7579 |
| **Externalizing problems** | **Paternal education level** | ≤ Junior high school | **Dual non-employment** | 1.15 (0.92-1.44) | 0.2060 | 0.5650 |
| **Externalizing problems** | **Paternal education level** | High school diploma and junior college | **Father-only employment** | 0.79 (0.69-0.9) | 0.0006 | 0.0461 |
| **Externalizing problems** | **Paternal education level** | High school diploma and junior college | **Mother-only employment** | 1.45 (0.83-2.46) | 0.1806 | 0.5309 |
| **Externalizing problems** | **Paternal education level** | High school diploma and junior college | **Dual non-employment** | 1.14 (0.81-1.6) | 0.4430 | 0.7428 |
| **Externalizing problems** | **Paternal education level** | ≥ Undergraduate degree | **Father-only employment** | 1.06 (0.8-1.41) | 0.6776 | 0.8820 |
| **Externalizing problems** | **Paternal education level** | ≥ Undergraduate degree | **Mother-only employment** | 0.63 (0.09-2.69) | 0.5673 | 0.8379 |
| **Externalizing problems** | **Paternal education level** | ≥ Undergraduate degree | **Dual non-employment** | 1.05 (0.29-3.11) | 0.9386 | 0.9878 |
| **Externalizing problems** | **Paternal smoking status** | No | **Father-only employment** | 0.92 (0.81-1.04) | 0.1644 | 0.5209 |
| **Externalizing problems** | **Paternal smoking status** | No | **Mother-only employment** | 1.3 (0.79-2.08) | 0.2899 | 0.6325 |
| **Externalizing problems** | **Paternal smoking status** | No | **Dual non-employment** | 1.23 (0.97-1.54) | 0.0821 | 0.4030 |
| **Externalizing problems** | **Paternal smoking status** | Yes | **Father-only employment** | 0.89 (0.77-1.02) | 0.0829 | 0.4030 |
| **Externalizing problems** | **Paternal smoking status** | Yes | **Mother-only employment** | 1.18 (0.67-2) | 0.5501 | 0.8358 |
| **Externalizing problems** | **Paternal smoking status** | Yes | **Dual non-employment** | 0.97 (0.73-1.28) | 0.8308 | 0.9552 |
| **Externalizing problems** | **Paternal alcohol intake status** | No | **Father-only employment** | 0.91 (0.81-1.01) | 0.0814 | 0.4030 |
| **Externalizing problems** | **Paternal alcohol intake status** | No | **Mother-only employment** | 1.51 (0.98-2.3) | 0.0584 | 0.3676 |
| **Externalizing problems** | **Paternal alcohol intake status** | No | **Dual non-employment** | 1.11 (0.89-1.38) | 0.3345 | 0.6906 |
| **Externalizing problems** | **Paternal alcohol intake status** | Yes | **Father-only employment** | 0.89 (0.76-1.04) | 0.1509 | 0.5144 |
| **Externalizing problems** | **Paternal alcohol intake status** | Yes | **Mother-only employment** | 0.83 (0.4-1.59) | 0.5840 | 0.8455 |
| **Externalizing problems** | **Paternal alcohol intake status** | Yes | **Dual non-employment** | 1.12 (0.81-1.53) | 0.4829 | 0.7726 |
| **Externalizing problems** | **Maternal age, years** | <30 | **Father-only employment** | 1.01 (0.82-1.23) | 0.9489 | 0.9878 |
| **Externalizing problems** | **Maternal age, years** | <30 | **Mother-only employment** | 1.52 (0.54-3.96) | 0.3982 | 0.7216 |
| **Externalizing problems** | **Maternal age, years** | <30 | **Dual non-employment** | 1.93 (1.23-3.03) | 0.0042 | 0.1234 |
| **Externalizing problems** | **Maternal age, years** | 30-44 | **Father-only employment** | 0.88 (0.79-0.97) | 0.0118 | 0.1764 |
| **Externalizing problems** | **Maternal age, years** | 30-44 | **Mother-only employment** | 1.21 (0.81-1.79) | 0.3465 | 0.7066 |
| **Externalizing problems** | **Maternal age, years** | 30-44 | **Dual non-employment** | 1.02 (0.83-1.24) | 0.8757 | 0.9706 |
| **Externalizing problems** | **Maternal age, years** | ≥45 | **Father-only employment** | 1.15 (0.5-2.65) | 0.7384 | 0.9036 |
| **Externalizing problems** | **Maternal age, years** | ≥45 | **Mother-only employment** | 0.86 (0.1-5.35) | 0.8786 | 0.9706 |
| **Externalizing problems** | **Maternal age, years** | ≥45 | **Dual non-employment** | 0.78 (0.24-2.38) | 0.6640 | 0.8732 |
| **Externalizing problems** | **Maternal education level** | ≤ Junior high school | **Father-only employment** | 0.92 (0.79-1.06) | 0.2545 | 0.6175 |
| **Externalizing problems** | **Maternal education level** | ≤ Junior high school | **Mother-only employment** | 1.19 (0.56-2.39) | 0.6392 | 0.8588 |
| **Externalizing problems** | **Maternal education level** | ≤ Junior high school | **Dual non-employment** | 1.09 (0.86-1.36) | 0.4870 | 0.7748 |
| **Externalizing problems** | **Maternal education level** | High school diploma and junior college | **Father-only employment** | 0.89 (0.79-1.01) | 0.0693 | 0.3995 |
| **Externalizing problems** | **Maternal education level** | High school diploma and junior college | **Mother-only employment** | 1.16 (0.68-1.91) | 0.5727 | 0.8397 |
| **Externalizing problems** | **Maternal education level** | High school diploma and junior college | **Dual non-employment** | 1.14 (0.82-1.56) | 0.4258 | 0.7408 |
| **Externalizing problems** | **Maternal education level** | ≥ Undergraduate degree | **Father-only employment** | 0.86 (0.62-1.17) | 0.3392 | 0.6965 |
| **Externalizing problems** | **Maternal education level** | ≥ Undergraduate degree | **Mother-only employment** | 1.42 (0.68-2.87) | 0.3323 | 0.6897 |
| **Externalizing problems** | **Maternal education level** | ≥ Undergraduate degree | **Dual non-employment** | 2.02 (0.68-5.53) | 0.1795 | 0.5309 |
| **Externalizing problems** | **Maternal smoking status** | No | **Father-only employment** | 0.9 (0.82-0.99) | 0.0288 | 0.2572 |
| **Externalizing problems** | **Maternal smoking status** | No | **Mother-only employment** | 1.27 (0.87-1.83) | 0.2010 | 0.5553 |
| **Externalizing problems** | **Maternal smoking status** | No | **Dual non-employment** | 1.14 (0.95-1.36) | 0.1615 | 0.5209 |
| **Externalizing problems** | **Maternal smoking status** | Yes | **Father-only employment** | 0.93 (0.55-1.53) | 0.7755 | 0.9303 |
| **Externalizing problems** | **Maternal smoking status** | Yes | **Mother-only employment** | 1.02 (0.14-4.88) | 0.9783 | 0.9886 |
| **Externalizing problems** | **Maternal smoking status** | Yes | **Dual non-employment** | 0.64 (0.17-1.98) | 0.4693 | 0.7669 |
| **Externalizing problems** | **Maternal alcohol intake status** | No | **Father-only employment** | 0.89 (0.81-0.98) | 0.0168 | 0.2081 |
| **Externalizing problems** | **Maternal alcohol intake status** | No | **Mother-only employment** | 1.25 (0.86-1.79) | 0.2328 | 0.5960 |
| **Externalizing problems** | **Maternal alcohol intake status** | No | **Dual non-employment** | 1.12 (0.93-1.34) | 0.2147 | 0.5776 |
| **Externalizing problems** | **Maternal alcohol intake status** | Yes | **Father-only employment** | 1.22 (0.78-1.9) | 0.3760 | 0.7216 |
| **Externalizing problems** | **Maternal alcohol intake status** | Yes | **Mother-only employment** | 1.39 (0.18-7.69) | 0.7182 | 0.9036 |
| **Externalizing problems** | **Maternal alcohol intake status** | Yes | **Dual non-employment** | 1.09 (0.33-3.25) | 0.8799 | 0.9706 |
| **Prosocial behavior** | **Child age, years** | <4 | **Father-only employment** | 0.95 (0.8-1.12) | 0.5089 | 0.7976 |
| **Prosocial behavior** | **Child age, years** | <4 | **Mother-only employment** | 1.49 (0.59-3.82) | 0.3963 | 0.7216 |
| **Prosocial behavior** | **Child age, years** | <4 | **Dual non-employment** | 0.77 (0.51-1.16) | 0.2156 | 0.5776 |
| **Prosocial behavior** | **Child age, years** | 4-5 | **Father-only employment** | 0.98 (0.85-1.12) | 0.7479 | 0.9088 |
| **Prosocial behavior** | **Child age, years** | 4-5 | **Mother-only employment** | 0.85 (0.49-1.46) | 0.5587 | 0.8358 |
| **Prosocial behavior** | **Child age, years** | 4-5 | **Dual non-employment** | 0.89 (0.66-1.2) | 0.4506 | 0.7490 |
| **Prosocial behavior** | **Child age, years** | ≥5 | **Father-only employment** | 1.09 (0.97-1.21) | 0.1442 | 0.5144 |
| **Prosocial behavior** | **Child age, years** | ≥5 | **Mother-only employment** | 0.92 (0.57-1.47) | 0.7228 | 0.9036 |
| **Prosocial behavior** | **Child age, years** | ≥5 | **Dual non-employment** | 0.95 (0.76-1.18) | 0.6205 | 0.8588 |
| **Prosocial behavior** | **Child gender** | Boys | **Father-only employment** | 1.07 (0.97-1.19) | 0.1859 | 0.5390 |
| **Prosocial behavior** | **Child gender** | Boys | **Mother-only employment** | 0.91 (0.58-1.41) | 0.6826 | 0.8841 |
| **Prosocial behavior** | **Child gender** | Boys | **Dual non-employment** | 1.05 (0.84-1.31) | 0.6631 | 0.8732 |
| **Prosocial behavior** | **Child gender** | Girls | **Father-only employment** | 0.97 (0.87-1.08) | 0.5417 | 0.8358 |
| **Prosocial behavior** | **Child gender** | Girls | **Mother-only employment** | 0.96 (0.58-1.61) | 0.8736 | 0.9706 |
| **Prosocial behavior** | **Child gender** | Girls | **Dual non-employment** | 0.74 (0.59-0.94) | 0.0144 | 0.1843 |
| **Prosocial behavior** | **Marital status** | Married/Living with partner | **Father-only employment** | 1.03 (0.95-1.11) | 0.4965 | 0.7846 |
| **Prosocial behavior** | **Marital status** | Married/Living with partner | **Mother-only employment** | 0.86 (0.61-1.22) | 0.4019 | 0.7216 |
| **Prosocial behavior** | **Marital status** | Married/Living with partner | **Dual non-employment** | 0.9 (0.76-1.06) | 0.1921 | 0.5432 |
| **Prosocial behavior** | **Marital status** | Others | **Father-only employment** | 0.8 (0.45-1.41) | 0.4298 | 0.7408 |
| **Prosocial behavior** | **Marital status** | Others | **Mother-only employment** | 2.73 (0.83-10.82) | 0.1173 | 0.4950 |
| **Prosocial behavior** | **Marital status** | Others | **Dual non-employment** | 0.82 (0.39-1.72) | 0.5955 | 0.8468 |
| **Prosocial behavior** | **Annual family income, in thousands, RMB** | < 30 | **Father-only employment** | 1.03 (0.92-1.15) | 0.6438 | 0.8588 |
| **Prosocial behavior** | **Annual family income, in thousands, RMB** | < 30 | **Mother-only employment** | 0.9 (0.6-1.34) | 0.6035 | 0.8468 |
| **Prosocial behavior** | **Annual family income, in thousands, RMB** | < 30 | **Dual non-employment** | 0.89 (0.74-1.08) | 0.2348 | 0.5971 |
| **Prosocial behavior** | **Annual family income, in thousands, RMB** | ≥ 30 to 100 | **Father-only employment** | 1.04 (0.92-1.17) | 0.5553 | 0.8358 |
| **Prosocial behavior** | **Annual family income, in thousands, RMB** | ≥ 30 to 100 | **Mother-only employment** | 0.99 (0.53-1.86) | 0.9771 | 0.9886 |
| **Prosocial behavior** | **Annual family income, in thousands, RMB** | ≥ 30 to 100 | **Dual non-employment** | 0.83 (0.59-1.17) | 0.2812 | 0.6261 |
| **Prosocial behavior** | **Annual family income, in thousands, RMB** | ≥ 100 | **Father-only employment** | 0.94 (0.74-1.18) | 0.5766 | 0.8397 |
| **Prosocial behavior** | **Annual family income, in thousands, RMB** | ≥ 100 | **Mother-only employment** | 0.75 (0.09-6.55) | 0.7807 | 0.9310 |
| **Prosocial behavior** | **Annual family income, in thousands, RMB** | ≥ 100 | **Dual non-employment** | 2.44 (0.8-9.1) | 0.1393 | 0.5144 |
| **Prosocial behavior** | **Depression risk** | No | **Father-only employment** | 1 (0.92-1.09) | 0.9507 | 0.9878 |
| **Prosocial behavior** | **Depression risk** | No | **Mother-only employment** | 0.79 (0.54-1.18) | 0.2502 | 0.6120 |
| **Prosocial behavior** | **Depression risk** | No | **Dual non-employment** | 0.88 (0.73-1.05) | 0.1627 | 0.5209 |
| **Prosocial behavior** | **Depression risk** | Yes | **Father-only employment** | 1.11 (0.91-1.35) | 0.2932 | 0.6355 |
| **Prosocial behavior** | **Depression risk** | Yes | **Mother-only employment** | 1.41 (0.76-2.62) | 0.2740 | 0.6261 |
| **Prosocial behavior** | **Depression risk** | Yes | **Dual non-employment** | 0.94 (0.67-1.31) | 0.7054 | 0.8936 |
| **Prosocial behavior** | **Paternal age, years** | <30 | **Father-only employment** | 1.03 (0.79-1.33) | 0.8368 | 0.9592 |
| **Prosocial behavior** | **Paternal age, years** | <30 | **Mother-only employment** | 1.14 (0.36-3.96) | 0.8244 | 0.9533 |
| **Prosocial behavior** | **Paternal age, years** | <30 | **Dual non-employment** | 0.97 (0.48-1.96) | 0.9338 | 0.9878 |
| **Prosocial behavior** | **Paternal age, years** | 30-44 | **Father-only employment** | 1.02 (0.94-1.11) | 0.5586 | 0.8358 |
| **Prosocial behavior** | **Paternal age, years** | 30-44 | **Mother-only employment** | 0.81 (0.56-1.17) | 0.2666 | 0.6235 |
| **Prosocial behavior** | **Paternal age, years** | 30-44 | **Dual non-employment** | 0.88 (0.74-1.05) | 0.1655 | 0.5209 |
| **Prosocial behavior** | **Paternal age, years** | ≥45 | **Father-only employment** | 1.03 (0.69-1.56) | 0.8692 | 0.9705 |
| **Prosocial behavior** | **Paternal age, years** | ≥45 | **Mother-only employment** | 2.01 (0.64-7.68) | 0.2577 | 0.6185 |
| **Prosocial behavior** | **Paternal age, years** | ≥45 | **Dual non-employment** | 0.94 (0.51-1.74) | 0.8482 | 0.9608 |
| **Prosocial behavior** | **Paternal education level** | ≤ Junior high school | **Father-only employment** | 0.95 (0.84-1.07) | 0.3999 | 0.7216 |
| **Prosocial behavior** | **Paternal education level** | ≤ Junior high school | **Mother-only employment** | 0.88 (0.55-1.41) | 0.6001 | 0.8468 |
| **Prosocial behavior** | **Paternal education level** | ≤ Junior high school | **Dual non-employment** | 0.83 (0.68-1.02) | 0.0752 | 0.4030 |
| **Prosocial behavior** | **Paternal education level** | High school diploma and junior college | **Father-only employment** | 1.05 (0.94-1.17) | 0.4290 | 0.7408 |
| **Prosocial behavior** | **Paternal education level** | High school diploma and junior college | **Mother-only employment** | 0.88 (0.53-1.46) | 0.6119 | 0.8513 |
| **Prosocial behavior** | **Paternal education level** | High school diploma and junior college | **Dual non-employment** | 0.98 (0.72-1.34) | 0.9217 | 0.9878 |
| **Prosocial behavior** | **Paternal education level** | ≥ Undergraduate degree | **Father-only employment** | 1.21 (0.95-1.55) | 0.1308 | 0.5144 |
| **Prosocial behavior** | **Paternal education level** | ≥ Undergraduate degree | **Mother-only employment** | 1.71 (0.43-7.17) | 0.4407 | 0.7428 |
| **Prosocial behavior** | **Paternal education level** | ≥ Undergraduate degree | **Dual non-employment** | 0.57 (0.19-1.57) | 0.2830 | 0.6261 |
| **Prosocial behavior** | **Paternal smoking status** | No | **Father-only employment** | 1.04 (0.94-1.16) | 0.3983 | 0.7216 |
| **Prosocial behavior** | **Paternal smoking status** | No | **Mother-only employment** | 0.97 (0.62-1.51) | 0.8879 | 0.9721 |
| **Prosocial behavior** | **Paternal smoking status** | No | **Dual non-employment** | 0.98 (0.79-1.21) | 0.8267 | 0.9533 |
| **Prosocial behavior** | **Paternal smoking status** | Yes | **Father-only employment** | 0.99 (0.89-1.12) | 0.9248 | 0.9878 |
| **Prosocial behavior** | **Paternal smoking status** | Yes | **Mother-only employment** | 0.89 (0.53-1.46) | 0.6332 | 0.8588 |
| **Prosocial behavior** | **Paternal smoking status** | Yes | **Dual non-employment** | 0.79 (0.61-1.01) | 0.0612 | 0.3730 |
| **Prosocial behavior** | **Paternal alcohol intake status** | No | **Father-only employment** | 1.03 (0.94-1.12) | 0.5857 | 0.8455 |
| **Prosocial behavior** | **Paternal alcohol intake status** | No | **Mother-only employment** | 0.88 (0.59-1.33) | 0.5498 | 0.8358 |
| **Prosocial behavior** | **Paternal alcohol intake status** | No | **Dual non-employment** | 0.88 (0.72-1.07) | 0.1924 | 0.5432 |
| **Prosocial behavior** | **Paternal alcohol intake status** | Yes | **Father-only employment** | 1.01 (0.88-1.16) | 0.8962 | 0.9721 |
| **Prosocial behavior** | **Paternal alcohol intake status** | Yes | **Mother-only employment** | 1.01 (0.57-1.82) | 0.9598 | 0.9878 |
| **Prosocial behavior** | **Paternal alcohol intake status** | Yes | **Dual non-employment** | 0.92 (0.69-1.23) | 0.5773 | 0.8397 |
| **Prosocial behavior** | **Maternal age, years** | <30 | **Father-only employment** | 0.9 (0.75-1.07) | 0.2214 | 0.5776 |
| **Prosocial behavior** | **Maternal age, years** | <30 | **Mother-only employment** | 1.7 (0.66-4.7) | 0.2780 | 0.6261 |
| **Prosocial behavior** | **Maternal age, years** | <30 | **Dual non-employment** | 0.74 (0.47-1.16) | 0.1964 | 0.5465 |
| **Prosocial behavior** | **Maternal age, years** | 30-44 | **Father-only employment** | 1.05 (0.97-1.15) | 0.2306 | 0.5943 |
| **Prosocial behavior** | **Maternal age, years** | 30-44 | **Mother-only employment** | 0.81 (0.56-1.17) | 0.2557 | 0.6175 |
| **Prosocial behavior** | **Maternal age, years** | 30-44 | **Dual non-employment** | 0.91 (0.76-1.08) | 0.2753 | 0.6261 |
| **Prosocial behavior** | **Maternal age, years** | ≥45 | **Father-only employment** | 0.76 (0.37-1.55) | 0.4504 | 0.7490 |
| **Prosocial behavior** | **Maternal age, years** | ≥45 | **Mother-only employment** | 2.19 (0.39-17.81) | 0.4037 | 0.7216 |
| **Prosocial behavior** | **Maternal age, years** | ≥45 | **Dual non-employment** | 1.3 (0.5-3.48) | 0.5896 | 0.8458 |
| **Prosocial behavior** | **Maternal education level** | ≤ Junior high school | **Father-only employment** | 0.97 (0.86-1.1) | 0.6666 | 0.8736 |
| **Prosocial behavior** | **Maternal education level** | ≤ Junior high school | **Mother-only employment** | 0.65 (0.32-1.28) | 0.2182 | 0.5776 |
| **Prosocial behavior** | **Maternal education level** | ≤ Junior high school | **Dual non-employment** | 0.83 (0.67-1.02) | 0.0814 | 0.4030 |
| **Prosocial behavior** | **Maternal education level** | High school diploma and junior college | **Father-only employment** | 1.06 (0.95-1.17) | 0.2835 | 0.6261 |
| **Prosocial behavior** | **Maternal education level** | High school diploma and junior college | **Mother-only employment** | 1.02 (0.64-1.62) | 0.9371 | 0.9878 |
| **Prosocial behavior** | **Maternal education level** | High school diploma and junior college | **Dual non-employment** | 1.04 (0.78-1.38) | 0.7940 | 0.9390 |
| **Prosocial behavior** | **Maternal education level** | ≥ Undergraduate degree | **Father-only employment** | 1.01 (0.77-1.31) | 0.9583 | 0.9878 |
| **Prosocial behavior** | **Maternal education level** | ≥ Undergraduate degree | **Mother-only employment** | 1.17 (0.59-2.35) | 0.6462 | 0.8588 |
| **Prosocial behavior** | **Maternal education level** | ≥ Undergraduate degree | **Dual non-employment** | 0.26 (0.07-0.75) | 0.0200 | 0.2117 |
| **Prosocial behavior** | **Maternal smoking status** | No | **Father-only employment** | 1.03 (0.95-1.11) | 0.4725 | 0.7688 |
| **Prosocial behavior** | **Maternal smoking status** | No | **Mother-only employment** | 0.94 (0.67-1.32) | 0.7266 | 0.9036 |
| **Prosocial behavior** | **Maternal smoking status** | No | **Dual non-employment** | 0.89 (0.75-1.05) | 0.1588 | 0.5209 |
| **Prosocial behavior** | **Maternal smoking status** | Yes | **Father-only employment** | 0.93 (0.6-1.43) | 0.7346 | 0.9036 |
| **Prosocial behavior** | **Maternal smoking status** | Yes | **Mother-only employment** | 0.71 (0.15-3.19) | 0.6414 | 0.8588 |
| **Prosocial behavior** | **Maternal smoking status** | Yes | **Dual non-employment** | 1.28 (0.5-3.34) | 0.6081 | 0.8491 |
| **Prosocial behavior** | **Maternal alcohol intake status** | No | **Father-only employment** | 1.04 (0.96-1.13) | 0.2985 | 0.6404 |
| **Prosocial behavior** | **Maternal alcohol intake status** | No | **Mother-only employment** | 0.97 (0.69-1.36) | 0.8517 | 0.9619 |
| **Prosocial behavior** | **Maternal alcohol intake status** | No | **Dual non-employment** | 0.88 (0.75-1.04) | 0.1339 | 0.5144 |
| **Prosocial behavior** | **Maternal alcohol intake status** | Yes | **Father-only employment** | 0.64 (0.43-0.96) | 0.0326 | 0.2721 |
| **Prosocial behavior** | **Maternal alcohol intake status** | Yes | **Mother-only employment** | 0.48 (0.06-2.54) | 0.4040 | 0.7216 |
| **Prosocial behavior** | **Maternal alcohol intake status** | Yes | **Dual non-employment** | 2.08 (0.74-6.47) | 0.1811 | 0.5309 |
|  |  |  |  |  |  |  |

Adjusted model adjust for: all other stratification variables except the one being stratified

FDR = False Discovery Rate adjusted p-value.

Stratification variables were not involved in the adjustment of the respective stratification analysis.

All subgroup analyses were exploratory; FDR correction was applied to control multiple testing bias, significant interaction results need further replication.

**Table S3. Associations between parental employment status and emotional and behavioral problems in preschool children after imputing the baseline missing values**

| **Variables** | **Adjusted model** **before imputing**  **OR (95% CI) P value** | **Adjusted model after imputing**  **OR (95% CI) P value** |
| --- | --- | --- |
| **Total difficulties** |  |  |
| Dual-earner households | 1.0 | 1.0 |
| Father-only employment | 1.02 (0.93-1.12) 0.6545 | 1.26 (0.91-1.76) 0.1700 |
| Mother-only employment | 1.21 (0.85-1.7) 0.2752 | 1.02 (0.93-1.11) 0.6728 |
| Dual non-employment | 1.08 (0.9-1.29) 0.4032 | 1.09 (0.92-1.29) 0.3345 |
| **Internalizing problems** |  |  |
| Dual-earner households | 1.0 | 1.0 |
| Father-only employment | 1.11 (1.02-1.21) **0.0166** | 1 (0.71-1.4) 0.9884 |
| Mother-only employment | 0.97 (0.68-1.37) 0.8758 | 1.11 (1.02-1.21) **0.0117** |
| Dual non-employment | 1.31 (1.1-1.55) **0.0018** | 1.3 (1.1-1.52) **0.0015** |
| **Externalizing problems** |  |  |
| Dual-earner households | 1.0 | 1.0 |
| Father-only employment | 0.9 (0.83-0.98) **0.0126** | 1.19 (0.88-1.62) 0.2573 |
| Mother-only employment | 1.2 (0.87-1.65) 0.2551 | 0.9 (0.83-0.98) **0.0122** |
| Dual non-employment | 1.05 (0.89-1.24) 0.5383 | 1.05 (0.9-1.23) 0.5310 |
| **Prosocial behavior** |  |  |
| Dual-earner households | 1.0 | 1.0 |
| Father-only employment | 1.01 (0.94-1.09) 0.7494 | 1.03 (0.77-1.37) 0.8486 |
| Mother-only employment | 1.02 (0.75-1.37) 0.9185 | 1.02 (0.95-1.09) 0.6572 |
| Dual non-employment | 0.97 (0.83-1.12) 0.6738 | 1.01 (0.87-1.16) 0.9423 |
|  |  |  |
